# Calcareous Deposits

**Published:** 1883-08

**Authors:** L. C. Ingersoll

**Affiliations:** Keokuk, Iowa.


ARTICLE VII.

### CALCAREOUS DEPOSITS.

BY L. C. INGERSOLL, D. D. S., KEOKUK, IOWA.

During the few years past in which I have given some special attention to the presence and formation of calculus on the teeth, in obscure localities, I have become convinced that the profession are quite at fault as regards thoroughness in removing foreign deposits from the teeth of their patients who apply to them for this purpose; and further at fault in not appreciating the importance of the work.

I think that the majority of dentists usually think their operation finished when the salivary calculus has been removed from the exposed surfaces of the teeth, and the mouth presents a clean appearance.

But there is a delicate and far more important operation to be performed in most mouths, beneath the margin of the gum, in removing minute granules of sanguinary calculus which are deposited not from the saliva, but are a product of the decomposition of liquor sanguinis, which oozes from the capillary vessels, following congestion of the peridental membrane and the gum. This destructive inflammatory process is not induced solely by the deposit of salivary calculus upon the crowns of teeth, but may be induced by decomposing animal and vegetable matter that is lodged between the teeth and on the border line of the gums, and by the fetid sordes which collects on the teeth during the continuance of a general febrile condition, or gastric disturbance. The liquor sanguinis, which is the watery portion of the blood, holds in solution the lime salts which enter into the composition of the hard tissues of the body. When this fluid is decomposed, as in the formation of pus in thc alveoli, the lime salts are deposited in fine crystalline granules on the roots of the teeth.

The irritation, which is the starting-point of this diseased condition of the gum, may be nothing more than an excessive use of the brush in the daily or thrice daily cleansing of the teeth. Hence, we may find this suppurative condition and the resulting calcareous deposit on the roots of the teeth well cared for, and in the most cleanly mouths.

Not long since a lady whose teeth are exquisitely well kept, the teeth of a pearl whiteness and the gums of a rosy pink color, called my attention to a red spot about the size of a pin's head and located just below the margin of the gum, overlying the root of the left inferior lateral incisor, and another spot even, just below that. To me it was sufficient indication of the presence of sanguinary calculus. On examination I found the gum detached from the tooth, and in passing a very small and thin excavator down behind the reddened spot, I found a minute granule of calculus scarcely larger than a pin's point, and still below it another behind the second red spot. On applying the test for the presence of pus, I fouud it in small quantity. I examined two other teeth and found a similar condition.

If in a mouth so healthy and the teeth so well cleansed, I found a deposit of calculus, what must be the condition in mouths only tolerably well kept?—not to mention mouths in a state of evident neglect.

I have no doubt that with a little experience in making the search, a careful examination would reveal granules of tartar on the lateral sides of the roots of some teeth in nearly every mouth, and that we should find also, though possibly in a very slight degree, that condition which uniformly precedes the deposit of this form of calculus, namely, ulceration. This is the feeble beginning of a glaring, staring, recession of the gum, overlying one, two, three, or more teeth—a condition which usually passes unnoticed, both by the patient and the dentist, until the frightful recession has taken place.—*Missouri Dental Journal.*

